# Risk and clinical characteristics of spinal cord compression across different mucopolysaccharidosis types: A retrospective cohort study

**DOI:** 10.1097/MD.0000000000040113

**Published:** 2024-10-18

**Authors:** Insung Kim, Juyoung Sung, Yoon Ji Ahn, Minji Im, Min-Ji Kim, Se-Jun Park, Sung Yoon Cho

**Affiliations:** a Department of Pediatrics, Samsung Medical Center, Sungkyunkwan University School of Medicine, Seoul, Korea; b Biomedical Statistics Center, Research Institute for Future Medicine, Samsung Medical Center, Seoul, Korea; c Department of Orthopedic Surgery, Samsung Medical Center, Sungkyunkwan University School of Medicine, Seoul, Korea

**Keywords:** enzyme replacement therapy, kyphoscoliosis, ligament thickening, mucopolysaccharidosis, skeletal dysplasia, spinal cord compression

## Abstract

In patients with mucopolysaccharidosis (MPS), the accumulation of glycosaminoglycans leads to various complications, including spinal cord compression (SCC). Although SCC is a well-known complication in MPS, data comparing its clinical features across different MPS types remain limited. This study aimed to investigate the timing, location, and underlying causes of SCC in MPS, as well as to compare the risk and clinical characteristics by MPS type. We conducted a retrospective cohort study, reviewing the medical records of 183 patients with all types of MPS who were followed at Samsung Medical Center from January 1995 to March 2024. The distribution of patients diagnosed with SCC by MPS type was 33.3% for type I, 10.5% for type II, 55.0% for type IV, and 100% for type VI. The median age at SCC diagnosis was 16.3 years. Compared to type II, the risk of SCC was higher for type I (2.4 times, 95% confidence interval [CI]: 0.9–6.2), type IV (3.5 times; 95% CI: 1.5–8.1), and type VI (4.5 times, 95% CI: 1.2–16.4). Enzyme replacement therapy did not reduce the risk of SCC (*P* = .70). Moreover, SCC most frequently occurred at the C0 to C4 and T11 to L2 spinal levels. In the cervical spine, ligament thickening, and skeletal deformities were the predominant causes, whereas in the thoracolumbar spine, kyphoscoliosis and intervertebral disc issues were the main contributors. Although there was no significant difference in the location of SCC (*P* = .99), the underlying causes varied significantly by MPS type (*P* < .001). SCC is a common complication in MPS, but its risk and pathophysiology vary by MPS type. Therefore, an individualized approach is needed for early detection and appropriate intervention.

## 1. Introduction

Mucopolysaccharidosis (MPS) is a lysosomal storage disorder caused by the defective activity of specific lysosomal enzymes responsible for degrading glycosaminoglycans (GAGs). The disease is currently classified into 7 types (I, II, III, IV, VI, VII, and IX) based on the defective genes and enzymes involved. Type II follows an X-linked recessive inheritance pattern, while all other types are inherited in an autosomal recessive manner.^[1]^ The defective breakdown of GAGs leads to their accumulation within the lysosomes of various organs, including the eyes, central nervous system, skeletal system, nervous system, respiratory system, cardiac system, and gastrointestinal tract. The clinical severity of MPS varies both within and between types.^[2–7]^

Enzyme replacement therapy (ERT) is currently available for types I, II, IVA, VI, and VII,^[8]^ while hematopoietic stem cell transplantation (HSCT) is an option for patients with all types except type III.^[9]^ Skeletal disease is common among patients with MPS, and spinal cord compression (SCC) is a well-known complication.^[10–14]^ SCC primarily arises from the thickening of ligaments around the spinal canal due to GAG accumulation, which gradually narrows the spinal canal. Structural abnormalities in the spinal bones, such as atlantoaxial instability and thoracolumbar kyphoscoliosis are also significant contributors to SCC in MPS.^[15,16]^

When SCC occurs in patients with MPS, it manifests as neurological pain, muscle weakness, and paralysis, all of which impair mobility and significantly reduce quality of life. Medical treatments such as ERT and HSCT seem to have limited impact on skeletal manifestations, including spinal involvement,^[17–22]^ and severe SCC often requires surgical intervention.

Most MPS patients are diagnosed during childhood, requiring continuous medical monitoring and treatment from an early age. Despite the established association between MPS and SCC, there has been a lack of research comparing the risk, location, and etiology of SCC across different MPS types. This study aims to analyze the age at diagnosis, the spinal levels affected, and the underlying mechanisms of SCC in MPS patients, and to investigate how the risk and clinical characteristics differ by MPS type.

## 2. Methods

### 2.1. Study design and patients

This retrospective open cohort study reviewed the medical histories and test results of patients with all types of MPS, confirmed through enzyme activity and genetic testing, who visited Samsung Medical Center from January 1995 to March 2024. This study was approved by the Institutional Review Board of Samsung Medical Center (approval number: 2024-06-003-002). Since the study posed minimal risk to subjects, the requirement for informed consent was waived. The study investigated the age at which target events occurred, from birth to the last follow-up. Data collected included patient age, sex, MPS type, age at initiation of ERT or HSCT, symptoms, motor and cognitive abilities, radiographic findings (including plain radiographs and spine magnetic resonance imaging [MRI]), and surgical records. Surgical intervention was recommended for patients when paralysis occurred or was deemed imminent based on the spine surgeon’s assessment and radiographic findings, such as increased signal intensity in nerves on T2-weighted MRI images, severe craniocervical instability, or pronounced kyphoscoliosis. Surgery was performed when the patient’s condition permitted.

### 2.2. State definition

In this study, patients with SCC were defined as those presenting with symptoms of pain, muscle weakness, or paralysis, with spinal canal stenosis affecting the spinal cord confirmed through radiographic findings (effacement of cerebrospinal fluid in all directions around the spinal canal or increased signal intensity in nerves on T2-weighted MRI images) and examination by a spine surgeon.

The main etiology of lesions was classified into 4 categories based on radiographic findings: ligament thickening, skeletal dysplasia (any manifestation of dysostosis multiplex associated with MPS, excluding kyphoscoliosis), kyphoscoliosis, and intervertebral disc problems (such as protrusion or herniation).

An unevaluable state was defined as a condition in which a patient’s motor strength in all extremities was below grade 3, and they were unable to communicate sufficiently to assess pain or discomfort (i.e., bedridden state) or were deceased.

### 2.3. Data analysis

Data were analyzed using R software (version 4.3.2, R Core Team, Vienna, Austria). Basic statistics were expressed as medians, quartiles, and percentages. To analyze the age of SCC occurrence, 2 key steps were taken. First, survival analysis was conducted using Kaplan–Meier analysis to determine the point at which patients transitioned to an unevaluable state, considered the target event. This analysis aimed to determine how the evaluability of patients for SCC changed with age. Additionally, Kaplan–Meier survival analysis for SCC diagnosis was conducted, with the diagnosis of SCC as the target event and the transition to an unevaluable status before SCC diagnosis as a censoring event. This analysis focused on the age of SCC diagnosis in evaluable patients. Given the challenges of assessing SCC in patients who are immobile and/or unable to communicate, these patients were treated as censored prematurely to minimize bias that could suggest a lower incidence of SCC due to assessment difficulties. The analysis was stratified by MPS type to identify any differences between groups.

Additionally, this study aimed to determine whether receiving ERT had an impact on the timing of progression to an unevaluable state or the diagnosis of SCC. The ERT group included patients who received standard therapy with laronidase (Aldurazyme, Sanofi S.A., Paris, France, available in Korea since 2012) for type I, idursulfase (Elaprase, Sanofi S.A., Paris, France, available in Korea since 2013) or idursulfase beta (Hunterase, GC Biopharma, Yongin, Republic of Korea, available in Korea since 2012) for type II, elosulfase alfa (Vimizim, BioMarin Pharmaceutical Inc., California, USA, available in Korea since 2015) for type IVA, and galsulfase (Naglazyme, BioMarin Pharmaceutical Inc., available in Korea since 2008) for type VI. Patients who did not start ERT more than 6 months before the target or censoring event were classified into the untreated group. Patients with a history of HSCT were excluded from the analysis related to ERT status. The survival curve analysis was first performed using the log-rank test to evaluate differences in survival curves across MPS types or ERT status. Next, the hazard ratio and 95% confidence interval (CI) was derived using Cox proportional hazards regression after checking the proportional hazard assumption by Shoenfeld residual.

The frequency and primary etiology of spinal stenosis at different spine levels were analyzed using data from patients diagnosed with SCC. In cases where lesions occurred in multiple locations due to various causes in a single patient, the location and cause of each lesion were counted separately. Fisher exact test was used to determine whether there were statistically significant differences in the location and cause of SCC by type. For comparisons between individual types, Bonferroni correction was applied to control for inflation of the family-wise error rate. Additionally, the surgical techniques used on patients with SCC were summarized. In all analyses, *P* < .05 was considered to be statistically significant.

## 3. Results

This study included 183 patients diagnosed with MPS at Samsung Medical Center from January 1995 to March 2024. Among these patients, type II was the most common (105 patients, 57.4%), followed by type III (34 patients, 18.6%), type I (21 patients, 11.5%), type IV (20 patients, 10.9%), and type VI (3 patients, 1.6%). The median age of the patients at their last visit was 17.2 years (interquartile range (IQR): 11.5–22.5). A total of 119 patients (65.0%) received ERT, and 5 patients (2.7%) underwent HSCT. Three patients discontinued ERT during the study period (2 with type I and 1 with type II) and subsequently underwent HSCT. Thirty-two patients (17.5%) were diagnosed with SCC, 87 patients (47.5%) were evaluable and had no history of SCC at their last visit, and 64 patients (35.0%) were in an indeterminate state regarding SCC, as they were already unevaluable (Table 1).

**Table 1:** Clinical characteristics of patients with mucopolysaccharidoses.

	Type I	Type II	Type III	Type IV	Type VI	Total
N	21	105	34	20	3	183
Male, n (%)	11 (52.4)	104 (99.0)	21 (61.8)	9 (52.9)	1 (33.3)	146 (79.8)
Age* at the last visit	19.4 (10.5–26.4)	17.4 (11.3–21.4)	14.0 (11.6–16.6)	20.6 (16.9–26.7)	31.2 (26.5–36.7)	17.2 (11.5–22.5)
With ERT, n (%)	18 (85.7)	81 (77.1)	0 (0)	17 (85.0)	3 (100)	119 (65.0)
Age* at starting ERT	3.9 (2.3–11.7)	5.9 (3.5–15.0)	NA	10.1 (5.7–21.9)	15.7 (6.2–26.9)	6.2 (3.6–15.0)
HSCT, n (%)	2 (9.5)	3 (2.9)	0 (0)	0 (0)	0 (0)	5 (2.7)
SCC+, n (%)	7 (33.3)	11 (10.5)	0 (0)	11 (55.0)	3 (100)	32 (17.5)
Surgery+	6 (28.6)	2 (1.9)	NA	3 (15.0)	1 (33.3)	12 (6.6)
Surgery−	1 (4.8)	9 (8.6)	NA	8 (40.0)	2 (66.7)	20 (10.9)
SCC−, n (%)	13 (61.9)	53 (50.5)	13 (61.9)	8 (40.0)	0 (0)	87 (47.5)
Unevaluable status	1 (4.8)	41 (39.0)	21 (61.8)	1 (5.0)	0 (0)	64 (35.0)

% represents the proportion of each type.

ERT = enzyme replacement therapy, HSCT = hematopoietic stem cell transplantation, N = number, NA = not applicable, SCC = spinal cord compression.

* Age is expressed as the median (interquartile range).

**Figure 1.**
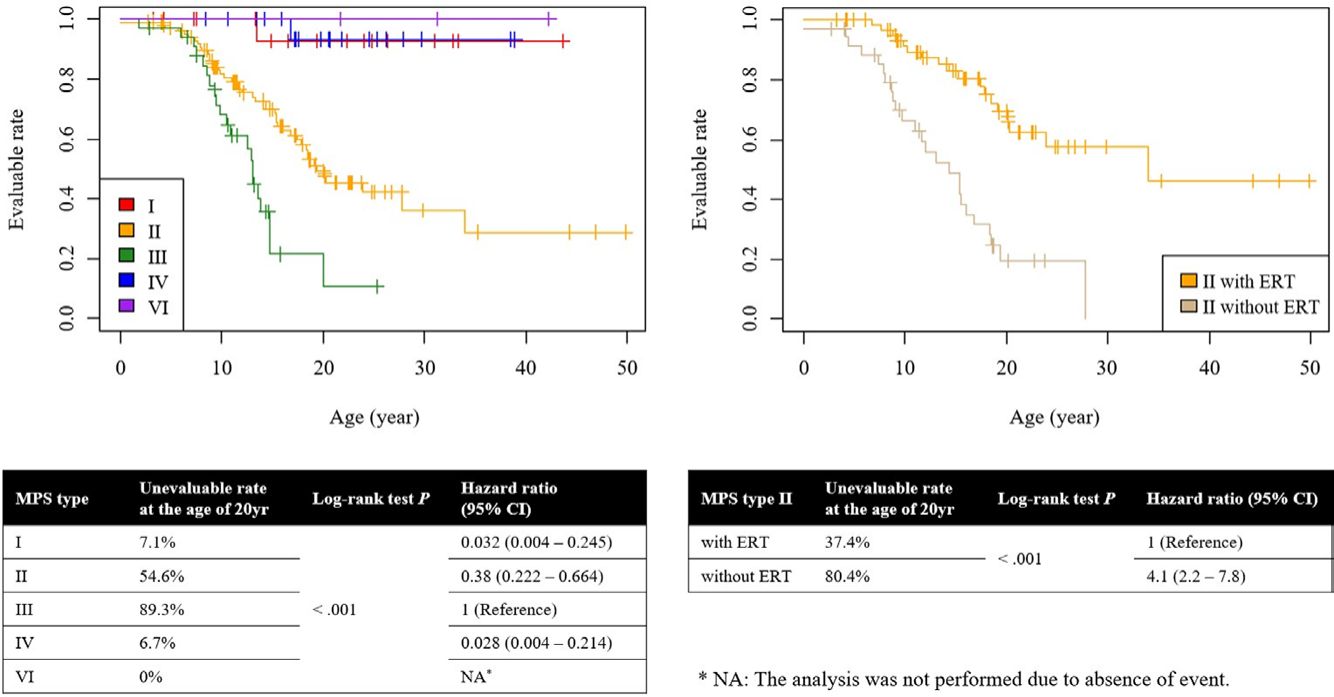
Survival analysis of unevaluable status by mucopolysaccharidosis type. MPS, mucopolysaccharidosis; CI, confidential interval; ERT, Enzyme Replacement Therapy.

In analyses investigating changes in the proportion of patients who remained evaluable for SCC over time, more than 90% of patients with MPS types I, IV, or VI were still evaluable after the age of 20 years. In contrast, only 45.4% of patients with type II and 10.7% with type III were evaluable at that age. The risk of becoming unevaluable was 0.032 times (95% CI: 0.004–0.245) lower for type I, 0.38 times (95% CI: 0.222–0.664) lower for type II, and 0.028 time (95% CI: 0.004–0.214) lower for type IV compared to type III. Among patients with MPS type II, who were at considerable risk of becoming unevaluable, those who received ERT were compared to those who did not. Of these, 62.6% of patients with ERT were still evaluable at the age of 20 years, while 80.4% of those without ERT had become unevaluable. The risk of becoming unevaluable without ERT was 4.1 times (95% CI: 2.2–7.8) higher than with ERT (Fig. 1). Among the 64 patients who became unevaluable, 68.8% (n = 44) gradually lost cognitive and motor functions due to degenerative neurological changes caused by MPS, while 17.2% (n = 11) suddenly became unevaluable due to acute respiratory failure (Table S1, Supplemental Digital Content, http://links.lww.com/MD/N760).

**Figure 2.**
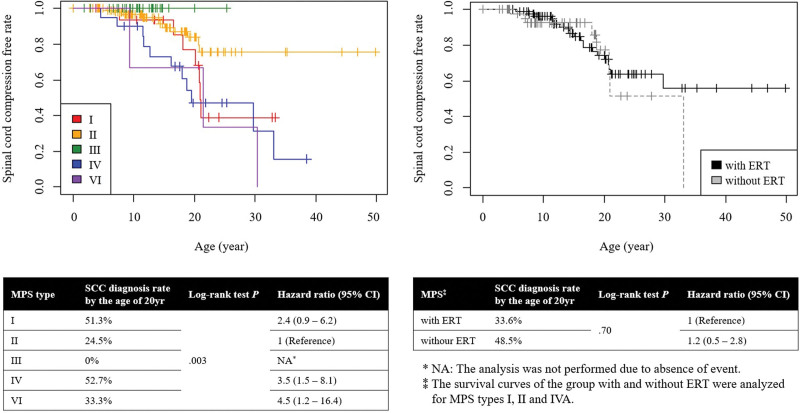
Survival analysis of spinal cord compression by mucopolysaccharidosis type. MPS: mucopolysaccharidosis; SCC, spinal cord compression; CI, confidential interval; ERT, enzyme replacement therapy.

We investigated the occurrence of SCC over time among evaluable patients by MPS type. For type II, 75.5% of patients had not developed SCC by the age of 20 years. Types I, IV, and VI showed similar Kaplan–Meier curve patterns, with a higher diagnosis rate of SCC around the age of 20 years, and the proportion of patients diagnosed with SCC increased over time. The risk of SCC was 2.4 times (95% CI: 0.9–6.2) higher for type I, 3.5 times (95% CI: 1.5–8.1) higher for type IV, and 4.5 times (95% CI: 1.2–16.4) higher for type VI compared to type II. No significant difference was observed in the Kaplan–Meier curves for SCC between the groups with and without ERT (*P* = .70) (patients with type III and type IVB, for whom no ERT was available, and patients with type VI, all of whom received ERT, were excluded from this analysis) (Fig. 2).

**Figure 3.**
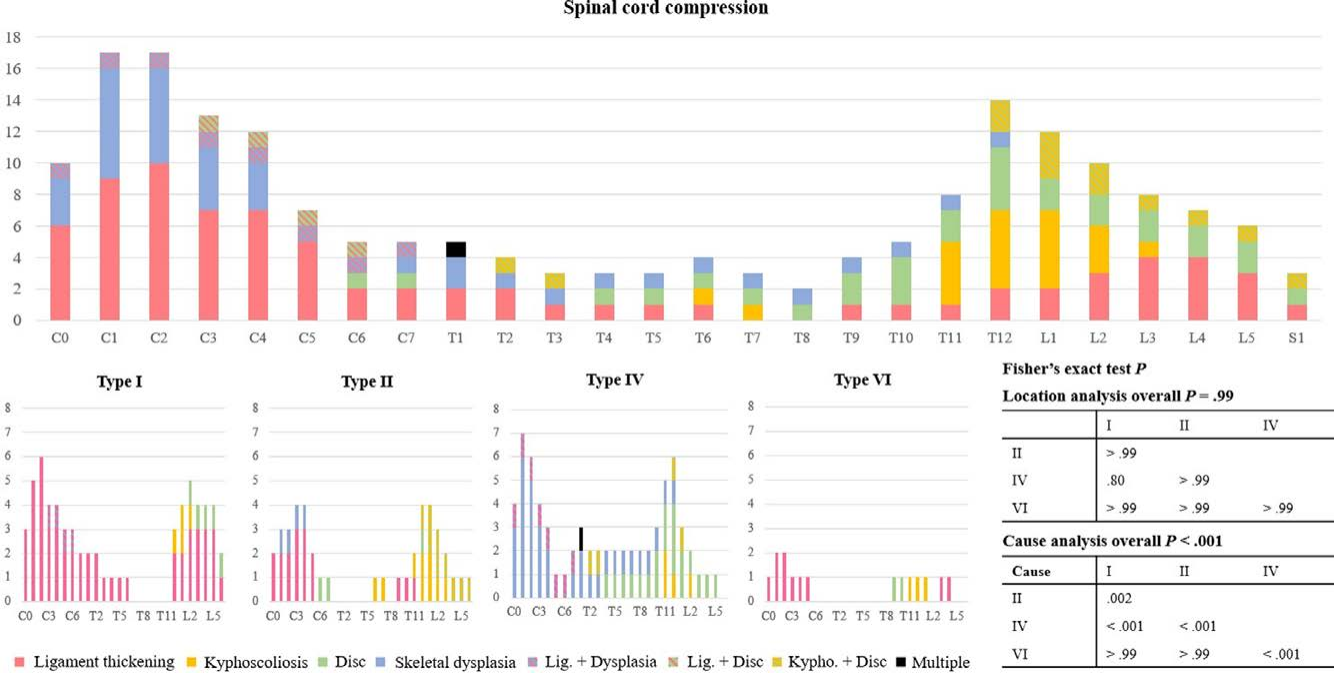
Analysis of the location and causes of spinal cord compression in patients with mucopolysaccharidosis.

When analyzing the locations and causes of SCC, spinal stenosis most frequently occurred at the C0 to C4 and T11 to L2 levels. In the cervical spine, the common causes were ligament thickening and skeletal deformities, while in the thoracolumbar spine, kyphoscoliosis and intervertebral disc herniation or protrusion were predominant. By MPS type, ligament thickening was the primary cause at all spinal locations in type I; in types II and VI, ligament thickening (cervical spine) and kyphoscoliosis (thoracolumbar spine) were predominant; and in type VI, skeletal deformities (cervical and thoracic spine) and disc problems (thoracic and lumbar spine) were most common. Although no significant difference was found in the location of spinal stenosis among the types (overall *P* = .99), there were significant differences in the causes of spinal stenosis between several types (overall *P* < .001) (Fig. 3).

The median age at SCC diagnosis was 16.3 years. Patients who underwent surgery were diagnosed earlier than those who did not (14.4 years vs 17.3 years). For those who had surgery, the median time from diagnosis to surgery was 1.1 years (Table 2). The surgical techniques performed on the patients were summarized by MPS type (Table 3).

**Table 2:** Ages at diagnosis and surgery in patients with spinal cord compression.

Patient group	Median (IQR) (yr)
All SCC + (n = 32)	
Age at diagnosis	16.3 (11.5–20.2)
SCC +, surgery − (n = 20)	
Age at diagnosis	17.3 (11.8–20.8)
SCC +, surgery + (n = 12)	
Age at diagnosis	14.4 (7.5–18.6)
Age at surgery	15.6 (11.3–19.2)
Time from diagnosis to surgery	1.1 (0.5–3.3)

IQR = interquartile range, MPS = mucopolysaccharidosis, SCC = spinal cord compression.

**Table 3:** Surgical techniques performed on patients with mucopolysaccharidoses for spinal cord compression.

MPS type	Surgical technique
I	6 Decompression of the cervical spinal canal
II	2 Thoracolumbar scoliosis correction
IV	1 Decompression of the cervical spinal canal 1 Stabilization of the cervical spine 1 Thoracolumbar kyphosis correction
VI	1 Thoracolumbar kyphosis correction and decompression of the spinal canal

MPS = mucopolysaccharidosis.

## 4. Discussion

This study provided a comprehensive analysis of the risk of SCC progression over time, the locations and causes of lesions, and the differences among MPS types. Analysis of the age at SCC diagnosis revealed that SCC was diagnosed at an earlier age and with a higher risk of occurrence in MPS types I, IV, and VI compared to type II. Notably, no patients with type III were diagnosed with SCC during the observation period. These findings are consistent with previous studies for each type.^[10–14,23,24]^

Guidelines recommend that patients with MPS types I, IV, or VI be monitored for SCC using simple X-ray imaging and MRI scans from the time of diagnosis.^[12,25–27]^ For MPS type II, cervical spine X-rays are essential, while lumbar spine X-rays and MRIs for abnormal spinal areas are optional.^[28,29]^ In type III, monitoring for scoliosis is necessary, but routine monitoring for cervical stenosis and instability is not recommended.^[23]^ As knowledge of MPS has increased and newborn screening has been introduced, the age of diagnosis has become younger, with many patients now being diagnosed in infancy or early childhood.^[30]^ In this context, the need for MRI scans must be carefully considered, given their cost and the need for sedation in young or noncooperative patients. In this study, SCC was predominantly diagnosed in teenagers, with the youngest patients diagnosed at ages 7.6 years for type I, 5.4 years for type II, 4.6 years for type IV, and 9.3 years for type VI. Based on these findings, we suggest that MPS patients diagnosed at a young age and without symptoms of SCC undergo initial collaborative monitoring by a physician and spine surgeon. Following this, MRI scans should be considered starting at age 5 and performed regularly every 1 to 2 years from age 10, if feasible. Moreover, it is crucial to tailor examinations and tests to each individual patient.

In this study, while there was no significant difference in the location of SCC across MPS types, significant differences were observed in the underlying causes of SCC. Guidelines for monitoring SCC in patients with MPS emphasize the importance of paying particular attention to stenosis and instability of the cervical spine. Consistent with these guidelines, many surgical cases in this study involved the cervical spine. However, the specific causes of SCC varied within the cervical spine itself: in MPS type IV, skeletal dysplasia was the predominant factor, whereas ligament thickening was the primary cause in MPS type I. Additionally, thoracolumbar kyphoscoliosis and disc issues were identified as significant causes of SCC. These findings provide crucial insights into the pathophysiology of SCC that should be considered when monitoring patients and planning surgical interventions for each MPS type.

In patients with MPS, if muscle weakness, paralysis, gait disturbances, or intractable pain are attributed to SCC, or if prominent SCC is confirmed in imaging studies, surgical treatment should be considered.^[24,31]^ However, surgical intervention requires a multidisciplinary assessment of the overall medical condition, including factors such as upper airway narrowing, cervical skeletal deformities, and cardiopulmonary function.^[32,33]^ While surgery may be advantageous when focusing solely on SCC, there are instances where the risks may outweigh the benefits, particularly in patients who are challenging to manage under general anesthesia, medically unfit for surgery, or in a palliative care setting. Therefore, the timing of surgical intervention should be determined on an individual basis by experienced professionals in specialized centers.

The skeletal system has a relatively limited direct blood supply, leading to low penetration of ERT drugs, which explains why ERT has minimal effect on the skeletal manifestations of MPS.^[17–19,21]^ In this study, while ERT was effective in reducing the risk of patients becoming unevaluable, it did not lower the risk of SCC. Therefore, even in patients receiving ERT, regular monitoring of skeletal system manifestations, including the spine, remains essential. Given that neurological complications resulting from SCC significantly diminish quality of life, there is an urgent need for new therapeutic approaches that specifically target this debilitating aspect of the disease.

Additionally, the analysis of the point at which patients become unevaluable provides new and intriguing insights into when and why patients with MPS become entirely dependent on caregiver assistance from a clinical standpoint. In this study, most patients with MPS types I, IV, and VI remained evaluable for SCC even after reaching adulthood. However, approximately half of the patients with MPS type II and the majority of those with MPS type III became unevaluable before adulthood. Patients with MPS type II who did not receive ERT had a significantly higher risk of becoming unevaluable compared to those who received ERT, with their Kaplan–Meier curve resembling that of patients with MPS type III. The most common cause of an unevaluable state in MPS types II and III was chronic neurodegenerative changes due to MPS progression.

An analysis of the timing at which MPS patients reach an unevaluable state has not been previously conducted. Another study analyzing the survival period of MPS patients showed trends similar to those seen in the analysis of the unevaluable state in this study, indicating that survival probability decreases rapidly over time in MPS types II and III compared to MPS types I, IV, and VI.^[30]^ In a study analyzing survival based on ERT implementation in patients with MPS type II, those who received ERT had a 54% lower risk of death than those who did not.^[34]^ Given that respiratory or cardiac failure related to the underlying pathology of MPS is a common cause of death in these patients,^[2,34–36]^ and that current ERT has limited efficacy against the neurological complications of MPS,^[37]^ the similarity between the unevaluable state analysis in this study and survival period analysis in other studies is noteworthy. This suggests that the pathophysiology of MPS gradually affects the entire body rather than being confined to specific organs, with declines in cardiopulmonary function and neurological deterioration mutually influencing each other.

This study has a few limitations. First, the interpretation of the results may be limited due to the low incidence of MPS, varying incidence rates among types in Korea,^[38]^ the availability of treatments for each type, and differing treatment options depending on the time of diagnosis. In Korea, type II is the most common, while type VI is the rarest among the MPS types. In this study, there was also significant difference in the sample sizes for each MPS type, with type VI having a particularly small sample size, requiring caution when generalizing the results of its analysis. Second, the study defined patient symptoms as an essential criterion for diagnosing SCC. This may have led to an underestimation of SCC, as patients with prominent radiographic evidence of SCC might have mild or no symptoms, or their symptoms may not have been adequately expressed or recognized by caregivers. This is particularly relevant for types II and III, where a significant number of patients were already unevaluable, potentially leading to an underestimation of SCC. However, given the primary focus on the patient’s evaluable state, it is reasonable to conclude that the results reflect clinically significant cases of SCC. Lastly, the analysis did not consider the age at which ERT was initiated when determining the timing of an unevaluable state or SCC diagnosis. Patients who began ERT earlier may have had better outcomes. However, in our unreported analysis due to a small sample size, patients who started ERT at a younger age were at a higher risk of becoming unevaluable, likely because they had a more severe phenotype. A more detailed analysis considering the onset of ERT and phenotypic differences within each type would require a larger sample size, which may be challenging given the rarity of MPS.

This study provided comprehensive insights into the timing, locations, and causes of SCC in patients with MPS across different types. MPS types I, IV, and VI showed a higher risk of SCC compared to other types. Although the predominant locations of SCC were the cervical and thoracolumbar spine in all types, the underlying pathophysiological factors varied. SCC in patients with MPS progresses from childhood and cannot be prevented by ERT. Therefore, multidisciplinary care is essential to determine the appropriate timing and frequency of monitoring for SCC and when to consider surgical intervention, starting from diagnosis in the pediatric years.

## Acknowledgments

We thank our patients and their families, as well as all the clinical and research laboratory staff.

## Author contributions

**Conceptualization:** Insung Kim, Se-Jun Park, Sung Yoon Cho.

**Data curation:** Insung Kim, Juyoung Sung, Yoon Ji Ahn, Minji Im.

**Formal analysis:** Insung Kim, Min-Ji Kim.

**Investigation:** Insung Kim.

**Resources:** Insung Kim, Juyoung Sung, Yoon Ji Ahn, Minji Im.

**Supervision:** Se-Jun Park, Sung Yoon Cho.

**Visualization:** Insung Kim.

**Writing – original draft:** Insung Kim.

**Writing – review & editing:** Minji Im, Min-Ji Kim, Se-Jun Park, Sung Yoon Cho.

## Supplementary Material


