# Supplementary material for: Risk and clinical characteristics of spinal cord compression across different mucopolysaccharidosis types: A retrospective cohort study
Source: Medicine (Baltimore). 2024 Oct 18;103(42):e40113. doi: 10.1097/MD.0000000000040113 (PMC11495688; doi:10.1097/MD.0000000000040113)
Supplement: Supplementary file 1 [file medi-103-e40113-s001.docx]

**Supplementary Table 1** The causes of the patients with mucopolysaccharidoses becoming unevaluable for spinal cord compression

| Type | State | System | Progression | Cause | N |
| --- | --- | --- | --- | --- | --- |
| I | Deceased | Respiratory | Acute | Apnea | 1 |
| II | Bedridden | Neurological | Chronic | Neurodegenerative change due to MPS | 28 |
|  |  |  | Acute | Intracranial hemorrhage | 2 |
|  |  | Respiratory |  | Apnea | 1 |
|  |  |  |  | Food choking | 1 |
|  |  |  |  | Upper airway obstruction due to MPS | 1 |
|  | Deceased |  |  | Apnea | 1 |
|  |  |  |  | Bacterial pneumonia after BMT | 1 |
|  |  |  |  | Upper airway obstruction due to MPS | 3 |
|  |  | Unknown |  | Sudden death due to unknown direct cause | 3 |
| III | Bedridden | Neurological | Chronic | Neurodegenerative change due to MPS | 16 |
|  |  |  | Acute | Intracranial hemorrhage | 2 |
|  |  | Respiratory | Chronic | Lung inflammation due to chronic aspiration | 1 |
|  |  |  | Acute | Upper airway obstruction due to MPS | 1 |
|  | Deceased |  |  | Aspiration pneumonia | 1 |
| IVA | Bedridden | Respiratory | Chronic | Restrictive lung disease due to MPS | 1 |
| Total |  |  |  |  | 64 |

MPS, mucopolysaccharidosis
